# Metastatic hormone-naïve prostate cancer: a distinct biological entity

**DOI:** 10.1016/j.trecan.2024.06.005

**Published:** 2024-09

**Authors:** Jon Corres-Mendizabal, Francesca Zacchi, Natalia Martín-Martín, Joaquin Mateo, Arkaitz Carracedo

**Affiliations:** 1 Center for Cooperative Research in Biosciences (CIC bioGUNE), Basque Research and Technology Alliance (BRTA), Bizkaia Technology Park, 48160 Derio, Spain; 2 Section of Innovation Biomedicine-Oncology Area, Department of Engineering for Innovation Medicine (DIMI), University of Verona and University and Hospital Trust (AOUI) of Verona, Verona, Italy; 3 Vall Hebron Institute of Oncology (VHIO), Vall d'Hebron University Hospital Campus, Barcelona, Spain; 4 Centro de Investigación Biomédica en Red de Cáncer (CIBERONC), 28029 Madrid, Spain; 5 Translational Prostate Cancer Research Laboratory, CIC bioGUNE-Basurto, Biobizkaia Health Research Institute, 48903 Barakaldo, Bizkaia, Spain; 6 Ikerbasque, Basque Foundation for Science, Bilbao, Spain; 7 Biochemistry and Molecular Biology Department, University of the Basque Country (UPV/EHU), Bilbao, Spain

**Keywords:** prostate cancer, metastasis, clinical trials, molecular data, experimental models, metastatic hormone-naïve prostate cancer

## Abstract

Metastatic hormone-naïve prostate cancer (mHNPC) is often the initial form of presentation for metastatic prostate cancer and encompasses a heterogeneous patient population with high inter-patient heterogeneity in prognosis and response to therapy. A more precise treatment of mHNPC, guided by evidence-based biomarkers, remains an unmet medical need. In addition, the limited number of representative laboratory models of mHNPC hampers the translation of basic research into clinical applications. We provide a comprehensive overview of the clinical and biological features that characterize mHNPC, highlight molecular data that could explain the unique prognostic characteristics of mHNPC, and identify key open questions.

## Highlights

Patients with metastatic hormone-naïve prostate cancer (mHNPC) exhibit a uniquely aggressive clinical evolution.
The disease course and response to standard therapies of *de novo* mHNPC differ from those of other patient groups.
Clinical management in mHNPC benefits from intensification strategies that are distinct from those of localized or castration-resistant metastatic disease.
There is a limited understanding of the molecular alterations that lead to mHNPC.
There is a need to generate representative preclinical models to study mHNPC.

## Definition and key clinical and molecular characteristics of mHNPC

Prostate cancer (PCa) is the second most frequent cancer and the fifth leading cause of cancer death among men worldwide [1]. Most PCa-related deaths are associated with metastatic spread, a condition that can occur in a hormone-naïve or a castration-resistant setting. mHNPC refers to PCa that has spread to other organs beyond the prostate and has either not yet been treated with hormonal therapy (testosterone deprivation) or has been exposed to hormonal therapy but has grown again in the context of normal (non-castrate) testosterone levels. In medical literature, the terms metastatic castration-sensitive prostate cancer (mCSPC), metastatic castration-naïve prostate cancer (mCNPC), metastatic hormone-sensitive prostate cancer (mHSPC) and mHNPC are used interchangeably. Depending on whether metastatic disease is observed in parallel to an upfront diagnosis of PCa or in the form of relapsed disease after having received radical prostatectomy and/or radiation therapy, mHNPC is classified as '*de novo*' or 'relapsed', respectively. The relative incidence of *de novo* versus relapsed mHNPC depends on multiple factors and varies geographically. In Western countries the prevalence of metastatic disease is ~10% [1,2], with an even distribution between *de novo* and relapsed metastatic cases [3]. Much higher rates of *de novo* mHNPC are present in developing countries [4]. This higher incidence of *de novo* metastases could be explained, in part, by differences in early diagnosis and screening programs, access to novel imaging test modalities, and, in general, to inequalities in access to healthcare [5].

Mortality rates for PCa have decreased in most high-income countries since the mid-1990s as a result of key advances in early detection strategies and treatment options. However, *de novo* mHNPC continues to exhibit a high 5 year disease-specific mortality [4,6]. Moreover, after a decline in the use of prostate-specific antigen (PSA) testing in many countries, mHNPC incidence has spiked, and the decline in PCa mortality has leveled off [4., 5., 6., 7., 8.]. Therefore, the impact of 'early versus delayed diagnosis' introduces higher heterogeneity when considering mHNPC and challenges the identification of clinically relevant disease phenotypes based on biological differences.

Recent evidence suggests that *de novo* mHNPC has a distinct and more aggressive clinical trajectory than those cases where metastatic disease appears years after treatment of localized disease [9,10], such as shorter time to the development of castration resistance. In this review we discuss the current evidence on the clinical and molecular features that could underlie the distinct nature of mHNPC.

## mHNPC diagnosis

### Imaging tools

Definitive diagnosis of PCa is based on histological findings. Biopsies have traditionally been guided by ultrasound scans, although the advent of multiparametric magnetic resonance imaging (MRI) in recent years has increased the accuracy of diagnosis and disease stratification. Computed tomography (CT) and bone scanning (BS) are the standard techniques to complete the diagnosis of advanced PCa by evaluating the presence and extension of distant disease, and these remain the standard for assessing the efficacy of cancer therapies, as per PCWG3 (Prostate Cancer Clinical Trials Working Group 3) and RECIST (response evaluation criteria in solid tumors) version 1.1 [11,12].

New imaging techniques, such as whole-body MRI (WB-MRI), and radiolabeled prostate-specific membrane antigen (PSMA) and choline combined with positron emission tomography (PET), have significantly enhanced our ability to identify distant disease with higher sensitivity [13., 14., 15.]. Beyond the cost and accessibility issues that have limited widespread implementation of these techniques in routine clinical care, it should be noted that current evidence for managing patients with different states of PCa is mostly based on disease-staging definitions derived from CT and BS imaging. Incorporating more sensitive assays results in migration of patients traditionally considered to be non-metastatic to the metastatic group. Caution is required before extrapolating clinical evidence to these new disease-state definitions. Nevertheless, it is envisaged that these novel techniques will be progressively adopted in clinical practice, leading to a higher proportion of PCa being recognized as metastatic. With appropriate studies generating evidence of clinical utility, these assays will likely improve our accuracy to stratify patients with mHNPC in the near future.

Taken together, these imaging modalities have improved performance and accuracy in detecting and monitoring metastases, thus enabling more effective treatment strategies. However, additional research is necessary to establish their optimal utilization in clinical care.

### Solid tumor biopsy

Although histological evaluation of tumor biopsies remains the gold standard for PCa diagnosis, there is limited information about the existence of differential histological features in mHNPC, and there is no established histopathological stratification for mHNPC beyond the Gleason score. Only a handful of studies have explored specific differences from other pathological settings, such as PCa treated with hormonal therapy and castration-resistant PCa (CRPC). In this regard, distinguishable features include randomly arranged small glands, hyperchromatic nuclei, prominent nucleoli, the absence of a basal cell layer, and an increased number of apoptotic cells [16,17].

Genomic profiling of tissue biopsies has shown clinical value in late-stage mCRPC. However, limited accessibility to metastases and the lack of primary tumor resection in patients with mHNPC hinder sample availability, and thus present a challenge to molecular characterization studies in this clinical setting.

### Liquid biopsy

Liquid biopsy offers a less invasive alternative to solid biopsies, and enables molecular characterization and continuous monitoring [18]. Studies on mHNPC indicate that circulating tumor cells (CTCs) provide valuable information, complementing the data obtained from solid tumor biopsies. CTCs offer prognostic insights beyond radiographic disease volume owing to the possible limitations in the resolution of standard radiographic imaging [19., 20., 21.]. In addition, the development of gene expression-based signatures in CTCs may harbor further prognostic or predictive potential [21].

Cell-free DNA (cfDNA) and circulating tumor DNA (ctDNA) also represent powerful liquid-biopsy alternatives that can inform clinical decision-making through prognostic and predictive response and resistance biomarkers [22., 23., 24.]. The ctDNA fraction correlates with progression-free survival and overall survival in patients with mHNPC, and is proportional to the number of CTCs [25]. Studies report that 37–74% of these patients present detectable ctDNA [25., 26., 27., 28.]. Regarding mCRPC, it is uncertain whether ctDNA levels are comparable to those in mHNPC [28] or whether they increase significantly in patients with mCRPC [27]. Data supporting a reduction in the ctDNA fraction after androgen deprivation therapy (ADT) encourages its use for monitoring the response to treatment, although it implies that therapy could limit the acquisition of ctDNA for genomic characterization [25,27,28]. Interestingly, the utility of ctDNA could extend beyond disease monitoring because this parameter has been associated with visceral metastases [28], and a combination of ctDNA, disease volume, and alkaline phosphatase is better at stratifying patients according to overall survival than the individual variables [27].

## Current classification of mHNPC and differences in prognosis

There is currently no histopathological or molecular classification of mHNPC that guides patient stratification for management in clinical practice. However, different strategies previously validated in mCRPC are being tested in mHNPC clinical trials. Molecular stratification strategies include PARP inhibition based on homologous recombination deficiency profile, AKT inhibition in *PTEN-*deficient tumors, or stratification for LuPSMA ([^177^Lu]-prostate-specific membrane antigen-617 radionuclide) therapy based on PSMA expression. Clinical and pathological features based on disease burden [high volume (HV) vs low volume (LV)], time of metastasis presentation (*de novo* vs recurrent metastases), and location of metastases have also been used for patient stratification in clinical trials in the past decade [10,29., 30., 31., 32., 33.], supporting the validity of these classifications to guide therapeutic decisions. Despite the reported potential for stratification of the aforementioned parameters, survival differences between *de novo* and relapsed metastatic patients are still unclear because the different distributions of diagnostic PSA and time since diagnosis are confounding factors [34].

There are several molecular and clinical differences between *de novo* and recurrent mHNPC. Patients with *de novo* mHNPC typically exhibit higher median PSA levels at diagnosis compared to recurrent mHNPC [9,34]. In addition, they show lower hemoglobin and albumin values, a higher likelihood of lymph node metastasis, and a shorter duration of hormone sensitivity than patients with relapsed mHNPC [9]. Moreover, age was found as an independent predictor of shorter PCa-specific survival (PCSS) in men diagnosed with *de novo* mHNPC. Men aged ≥75 years experienced a mean PCSS at 5 years that was 6.7 months shorter than for men aged ≤54 years (95% CI 5.5–7.8 months) [35]. Further work will be necessary to determine the reason for poor outcomes in older men with *de novo* mHNPC [9,36]. Although it is unclear whether this is influenced by differences in tumor burden, these results collectively suggest that *de novo* and recurrent mHNPC are biologically distinct entities with different outcomes. A more profound understanding of the differences between these two mHNPC groups will require further molecular characterization studies in tissue biopsies.

## Molecular determinants of mHNPC

### Genomic features

The genomic characterization of mHNPC has been an intensive research focus in the past decade and the source of the majority of molecular data on mHNPC. PCa exhibits a low frequency of point mutations and is instead characterized by copy-number alterations and large-scale rearrangements [37,38]. However, no information regarding the profile of these alterations in mHNPC has been reported until very recently, and we still lack a comprehensive picture. Although some studies have claimed that the mutational burden in mHNPC is similar to that of locoregional tumors [27,39,40], others have placed mHNPC closer to mCRPC regarding genomic features [28,41,42]. Multi-region profiling of mHNPC depicted a genomic landscape similar to that observed in single-tissue biopsies from late-stage mCRPC [43]. These findings suggest that patients with mHNPC might have already developed many of the traits associated with aggressive disease before treatment exposure. Beyond the type of events that are observed in metastatic prostate tumors, we can speculate that the order in which genomic alterations accumulate in prostate cells could also play a role in the development of this lethal variant of PCa.

Common genetic alterations in mHNPC and mCRPC include *TP53* loss, Speckle-type POZ protein (*SPOP*) mutations, alterations in cell-cycle or DNA damage repair (DDR) genes, and WNT or PI3K pathway mutations (summarized in Table 1). The lack of mutations and amplifications in the androgen receptor (*AR*) gene in mHNPC represents a pivotal difference when comparing these two metastatic forms of PCa, presumably because these mutations in mCRPC can provide adaptive resistance to ADT and AR-targeting agents (ARTAs) [34,39,44,45]. Based on data from mCRPC precision medicine trials, this genomic profile supports the investigation of personalized or molecularly stratified treatment in mHNPC trials based on the unique molecular landscape of each patient [26., 27., 25.]. However, the acquisition of metastatic properties extends beyond the genomic features of the tumor. In this sense, transcriptional regulation, alternative RNA splicing, and reprogramming of the tumor microenvironment (TME) are also important contributing factors, but our understanding of mHNPC in this regard is still in its infancy.

**Table 1:** Genomic alterations in mHNPC^a^

Process/gene	Alteration	Outcome
Genome stability	Few point mutations [37,38] Large-scale genomic rearrangements and CNA [37,38]	Genomic alterations in mHNPC may align with those in mCRPC, despite conflicting reports [27,28,39,41., 42., 43.,^125^] Altered genome fraction correlates with disease volume, not metastasis time [34] Heterogeneity within and between different tumor foci and metastases [43] Prostate may have clonally independent cancers, with metastases containing distinct populations [43]
*AR*	Lack of *AR* mutations/amplifications [42,43]	*AR* aberrations commonly occur in mHNPC patients during ADT, likely signaling the transition to a castration-resistant stage [26,34,39,44,45,126]
*TP53*	Loss of *TP53*	Most frequent gene alteration in mHNPC [27,34,39,43,45,125,127] Early mutations in metastatic patients correlate with the number of metastatic lesions [39,45,127] *TP53* loss predicts progression-free survival and castration resistance better than AR [43,128] *TP53* mutations may outweigh disease volume in outcome determination [34,45,127] Might have a smaller impact on lung tropism [129]
*SPOP*	Inactivating mutations in *SPOP* [130]	Higher response rates to hormone therapy [34,131., 132., 133.] Similar or higher alteration frequencies than in mCRPC [39,42,43,129] Elevated *SPOP* alterations in recurrent and HV patients [34,42,45]
Cell cycle	*RB1* deletion or loss *CDK12* mutations	Alterations of cell-cycle genes are present in up to 16% of mHNPC patients [27,39,41., 42., 43.,^45^,^127^] Associated with a shorter time to castration resistance and worse overall survival [34,45,125] HV patients are more likely to have alterations in the cell cycle [34,127] The timing of metastases shows varied associations in different cohorts – some link cell-cycle changes to recurrent patients, others to *de novo* patients [34,45,127] *RB1* loss is the most common mutation and is linked to worse overall survival, especially when combined with the loss of other tumor-suppressor genes [27,39,42,45,125,134] CDK12 mutations are more prevalent in *de novo* patients [34,45]
DNA damage repair (DDR)	*BRCA2* and *CDK12* mutations are the most common	DDR pathway alterations correlate with PARP inhibitor response [44] 20–27% of mHNPC patients have DDR pathway mutations [39,42., 43., 44., 45.] Occurrence of DDR alterations is linked to disease volume [42,45,127] DDR pathway alterations may hasten castration resistance and worsen survival [27,34] There is a consensus that DDR pathway alterations do not increase with castration resistance, suggesting potential benefits from PARPi early in treatment [34,41,44] Patients with recurring metastases in the abdominal nodes, bones, or viscera, and patients with lung metastases, present higher DDR mutation rates [127,129] Mismatch repair gene alterations are also enriched in lung metastases, making these patients candidates for immune checkpoint blockade [39,44,129]
WNT pathway	Alterations in *APC*, *CTNNB1*, and *RNF43*	WNT pathway gene alterations occur in 10–20% of patients [27,39,42,45,135] WNT pathway mutations are more common in recurrences and HV disease [42,45,127] Reports conflict on the link between WNT pathway alterations and castration resistance development [34,39,45] WNT pathway mutations are associated with visceral and lung metastases [127,129,135]
PI3K pathway	*PTEN* inactivating mutations	PI3K pathway gene alterations correlate with more aggressive phenotype [44,136] Frequencies of PI3K pathway alterations are comparable in mHNPC and advanced PCa [39,42,43,45,125,129] Recurrent cases often exhibit PI3K pathway alterations [45] PI3K pathway alterations are unrelated to volume and prognosis [45] PTEN is commonly mutated in mHNPC and has been associated with worse prognosis, especially in combination with the loss of other tumor-suppressor genes [39,41., 42., 43.,^45^,^125^]

a Abbreviations: ADT, androgen deprivation therapy; AR, androgen receptor; CNA, copy-number alteration; HV, high volume disease; mCRPC, metastatic castration-resistant prostate cancer; mHNPC, metastatic hormone naïve prostate cancer; PARPi, PARP inhibitor; PCa, prostate cancer.

### Transcriptomic characteristics

The availability of high-throughput molecular data on mHNPC is progressively increasing, which will likely help to generate a more comprehensive molecular portrait of this aggressive disease. At present, however, the data on these patients often form part of larger datasets for which patients with mHNPC only constitute a small fraction and clinical annotations are often suboptimal [46,47]. These patients are generally not studied as an independent entity and are instead grouped with patients in other clinical states of PCa based on common clinical or pathological characteristics, although castration resistance is a major driver of PCa evolution. For instance, an extensive transcriptomics analysis of spine metastases that focused on the classification of metastases within their MetA-C subtype system included specimens from 15 patients with mHNPC [47], which were considered in the analysis without accounting for their different nature. Indeed, the study reported that these cases were enriched in the ADT-responsive subtype, but it is unclear how the presence of mHNPC in this group influenced the classification [47]. Another example pertains to a single-cell RNA sequencing analysis in PCa specimens, three of which were untreated and presented metastasis at the time of diagnosis, indicative of *de novo* hormone-naïve metastatic disease [46]. Although subsequent studies analyzing this dataset [48., 49., 50., 51., 52., 53., 54., 55., 56., 57.] did not account for the mHNPC nature of these specimens, reanalysis of this dataset based on patient metastatic status enabled the identification of distinct cellular programs in mHNPC [58].

In 2021, transcriptomic profiling of a subset of patients enrolled in the CHAARTED clinical trial [40] revealed that mHNPC exhibits lower AR activity than non-metastatic PCa at diagnosis and is enriched in basal or luminal B subtypes according to the PAM50 (prediction analysis of microarray 50) classifier [40]. Patients with mHNPC classified as luminal B benefited from ADT plus docetaxel combination therapy, while this combined treatment was not of benefit in cases classified as basal [40]. Interestingly, although the PAM50 classification was able to predict the benefit of combination therapy, classifying patients according to their AR activity was unable to do so [40]. Another study reported that patients with *de novo* mHNPC present lower AR activity than patients with recurrent metastases [59]. The CHAARTED patient cohort was enriched for patients with HV and *de novo* disease [40], and it remains possible that the lower AR signaling levels detected when comparing to patients with localized PCa were influenced by the inclusion of these patients. Therefore, although patients with *de novo* mHNPC exhibit lower AR activity than localized or recurrent patients, more tailored cohorts would need to be analyzed to investigate the status of recurrent mHNPC. It should be noted that the aforementioned study reported an improvement in overall survival for patients with mHNPC and low AR activity (the *de novo* patients in the cohort) when receiving ADT plus docetaxel, regardless of their PAM50 classification [59]. A recent preprint presented the most comprehensive transcriptional landscape of mHNPC side-by-side with localized PCa analyzed at the time at diagnosis [58], indicating that we will soon have at hand additional molecular data that will support a better molecular deconstruction of mHNPC, with implications for the clinical management of the disease.

### *AR* splice variants

Lower AR signaling could reflect tumor-intrinsic properties of low androgen requirements, in line with recent evidence that points to the existence of CRPC-like cells in untreated localized PCa [60,61]. Activating mutations or amplifications of *AR* are extremely rare in mHNPC. However, other molecular alterations that render these tumors insensitive to AR blockade could play a relevant role. Alternative splicing of the *AR* mRNA, leading to the expression of the AR-V7 isoform, is a mechanism to sustain ligand-independent AR signaling. The absence of the ligand-binding domain in AR-V7 renders the nuclear receptor active in the absence of androgens or in the presence of androgen signaling pathway inhibitors, resulting in more aggressive disease [62]. Different groups have explored the expression of AR-V7 in mHNPC, but with discrepant results that highlight the limitations in the detection of this isoform using different molecular methodologies [62., 63., 64.]. Because the cohorts studied to date are predominantly focused on localized PCa, definitive evidence concerning the frequency and abundance of AR-V7 in mHNPC remains to be provided.

### The tumor microenvironment

Previous research efforts have predominantly focused on the genomic aspects of the tumor cell compartment of mHNPC, limiting our knowledge of the mHNPC TME. Only a handful of studies have reported a higher abundance of CD163^+^ macrophages in metastatic tumors [65] or have characterized higher numbers of circulating cancer-associated fibroblasts (cCAFs) in mHNPC patient specimens [66]. In this regard, a recent study reported that (i) mHNPC metastases had more immune infiltration than localized tumors, (ii) immune infiltration correlated with response to therapy, (iii) immune infiltration varied depending on the localization of the metastases, (iv) ADT and anti-programmed cell death protein 1 (anti-PD-1) combination therapy caused an expansion of CD8^+^ T cells in metastasis sites, and (v) epithelial cell subcluster abundance changed after treatment [67]. This study alone suggests that the mHNPC TME might differ significantly from that of localized tumors, and that there are alterations in immune infiltration after ADT between the two clinical states [67]. In addition, it suggests that patients with mHNPC could benefit from an ADT plus immune checkpoint blockade therapy. Reported [46] and upcoming [58] single-cell RNA sequencing data will shed light on the qualitative and quantitative alterations in the TME of this aggressive disease.

## Locoregional PCa as a proxy for mHNPC

In the absence of more extensive molecular analysis of mHNPC, locoregional PCa (tumors with colonization of pelvic lymph nodes) can shed light on the molecular processes that underlie the acquisition of metastatic capacity in PCa, such as DNA methylation changes that occur in parallel to copy-number alterations across the clonal evolution of PCa [68]. In line with this notion, transcriptomic analysis highlighted the activation of oxidative phosphorylation in lymph node metastases [69], together with gene expression-based prognostic models in this disease [70]. Single-cell transcriptomic studies of lymph node metastases and LNCaP cells (a hormone-sensitive cell line derived from these lesions) concluded that (i) MYC plays an important role in metastatic progression, (ii) the TME at the metastatic sites is more immunosuppressive, and (iii) treatment resistance is linked to changes in chromatin accessibility [60,71]. However, it should be noted that the prognosis for patients with locoregional PCa is better than for mHNPC [72], and that these lymph node metastases might only recapitulate a fraction of the processes altered in tumors with these clinical features.

## Experimental modeling of mHNPC

Experimental systems are essential to provide causal evidence of the alterations that support and drive mHNPC pathogenesis, and to test the efficacy of potential therapeutic strategies. The limited research regarding this PCa setting and the accumulated clinical and molecular evidence in recent years imply that it is now a crucial moment to define and design models of mHNPC. Patient-derived xenografting (PDX) represents an attractive strategy to study tumors in an individualized manner because they faithfully recapitulate the tumor architecture and heterogeneity of each patient. However, xenografts are difficult to establish, and the lack of a competent immune system might pose limitations when translating the results to a clinical setting.

Hormone-sensitive PDX tumors have been established over the past decades. The LuCaP series was generated in the 1990s, and currently comprises >30 different PDX models and their derived organoids [73,74]. These models and several others are derived from metastatic samples and retain androgen sensitivity regardless of whether they come from treated [74,75] or untreated patients [74,76]. In addition, in the Melbourne Urological Research Alliance (MURAL) and MD Anderson Cancer Center (MDA) collections, some of the largest series of PCa PDX models, a minor fraction of the biopsies that generated the PDX models were from hormone-sensitive metastatic cases [77,78]. Although these models are a good starting point for mHNPC research, further efforts will be necessary to establish new PDX models that recapitulate the biology of metastatic disease in hormone-naïve conditions.

Other experimental PCa models retain specific characteristics of mHNPC for laboratory research and/or overcome some of the limitations of PDX models (Table 2). Cell lines are the most simplified model for studying cancer and are a powerful tool to understand specific aspects of the disease. However, the *in vivo* metastatic capacity of PCa cell lines is limited when they retain androgen sensitivity. Commercially available androgen-sensitive cell lines (e.g., LNCaP, LAPC-4, and VCaP) exhibit limited dissemination capacity, mostly restricted to lymph nodes [79., 80., 81., 82., 83.], and gain more pronounced metastatic potential as they acquire androgen-independent properties [84., 85., 86.]. Genetically engineered mouse models (GEMMs) offer advantages and limitations compared to cell lines and PDX. GEMMs enable the natural evolution of PCa to be studied from pathogenesis to dissemination in immunocompetent environments. However, as occurs in humans, aggressive murine models with metastatic capabilities are often accompanied by the development of castration resistance, even in unchallenged conditions [87., 88., 89., 90., 91., 92., 93., 94., 95., 96., 97., 98., 99.]. In line with this notion, the metastatic potential of hormone-sensitive mouse models, such as the transgenic adenocarcinoma mouse prostate (TRAMP) model, is only increased to clinically relevant levels after developing castration resistance [100,101]. Other currently available GEMMs with metastatic capacity that retain androgen sensitivity are based on the loss of tumor suppressor *Pten* [102,103] or the overexpression of proto-oncogene *Myc* [104,105]. The combination of both alterations results in metastasis to the liver, lungs, and bones while retaining sensitivity to castration [91,106].

**Table 2:** Summary of experimental models for studying mHNPC^a^

Model	Pros	Cons	Name	Origin	AR sensitivity	Metastatic capacity
Cell lines	Simple model Easy to manipulate Replicable Able to be integrated into a more complex model (e.g., xenografting)	Lack heterogeneity Lack 3D organization Not integrated into a complex system by default Low metastatic potential that increases as AR sensitivity decreases	LNCaP [79]	Lymph node met	Yes	No
			C4-2 [85]	Derived from LNCaP	Yes, but lower than parental LNCaP	LN/bones (OR) [84,86]
			LAPC-4 [80]	Lymph node met	Yes	Micrometastasis in hematopoietic tissue (SC) [80]
			VCaP [81]	Lumbar met	Yes (although derived from a hormone-refractory patient) [81]	Micrometastasis in lung (SC) [82] LN (OR) [83]
Patient-derived xenografts (PDX)	Recapitulate tumor architecture Retain heterogeneity Integrated into a complex model where they receive systemic input	Difficult to establish High maintenance No immune compartment	LuCaP series (>30 PDXs) [73,74]	Samples from met sites	Some (mostly come from treated patients)	Lungs (IT) [137] LN/lungs (OR) [138]
			Other PDXs (BM18, LAPC9, MURAL, MDA) [75., 76., 77., 78.]	Samples from met sites	Yes (some come from untreated patients) [76., 77., 78.]	Untested
Genetically engineered mouse models (GEMMs)	Allow the study of the evolution of PCa (from normal prostate to metastatic tumor) Best model to study the role of the immune compartment	High maintenance Lack the genomic heterogeneity seen in patients Genetic alterations performed to establish GEMMs may not be prevalent in the diseased population Metastatic potential often increases as AR sensitivity decreases [106., 107., 108., 109.]	TRAMP [100]	Introduction of prostate-specific SV40L	Yes	LN (low penetrance; higher metastatic potential after NE differentiation) [101]
			*Pten* [103]	Induced *Pten* loss (+ other optional mutations)	*Pten* loss: yes [103]*Pten* loss + TP53 loss: no [95,98,99]*Pten* loss + MAPK pathway activation: no [90,97]*Pten* loss + *Lkb1* loss: yes, but lower [93]*Pten* loss + *Pgc1a* loss: yes [102] *Pten* loss + *Kras* activation: no [91]	*Pten* loss: LN (low dissemination) [103] *Pten* loss + *Tp53* loss: visceral mets (when progressed to CRPC) [99] *Pten* loss + MAPK pathway activation: LN/bone/lungs [90,97] *Pten* loss + *Lkb1* loss: LN/lungs [93]*Pten* loss + *Pgc1a* loss: LN mets/bone micromets [102]*Pten* loss + *Kras* activation: lung/liver/bones [91,92,96]
			*Myc*	Induced *Myc* overexpression [104]	Yes	*Myc* overexpression GEMM: no [104] Allograft cell line derived from *Myc* overexpression GEMM: bone (IC) [105] *Myc* overexpression + *Pten* loss: liver/lung/bone [91,106]

a Abbreviations: AR, androgen receptor; CRPC, castration-resistant prostate cancer; GEMM, genetically engineered mouse model; IC, intracardiac injection; IT, intratibial injection; LN, lymph node; met, metastasis; NE, neuroendocrine; OR, orthotopic injection; PDX, patient-derived xenograft; SC, subcutaneous injection; TRAMP, transgenic adenocarcinoma mouse prostate model.

In conclusion, experimental models continue to be indispensable tools in advancing our understanding of mHNPC, but they present significant challenges. As research progresses, refining and rethinking experimental models of mHNPC will boost our knowledge of this aggressive disease and enhance their utility in guiding therapeutic strategies.

## Current treatments and clinical trials

ADT alone was the standard of care for mHNPC until 2015. The therapeutic landscape of mHNPC has rapidly evolved in recent years, marked by new data from landmark trials that endorse the intensification of upfront treatment. These advances extend beyond systemic agents and encompass innovations in treating primary tumors and metastases. A summary of the key clinical trials in the past decade can be found in Table 3.

**Table 3:** Key Phase 3 clinical trials including patients with HNPC^a^

Trial	Number of patients	Experimental arm	Control arm	*De novo* mHNPC	mOS (months if not otherwise specified)	HR (95% CI)	*P* value	Follow-up (months if not otherwise specified)
GETUG-AFU15 [31]	385	ADT + DTX	ADT	71%	Overall: 62.1 vs 48.6 Subgroup analysis HV: 39.8 vs 35.1 LV: NR vs 83.4 dn-HNPC: 52.6 vs 41.5 r-HNPC: NR vs 69,8	0.88 (0.68–1.14) 0.78 (0.56–1.09) 1.02 (0.67–1.55) 0.93 (0.69–1.25) 0.83 (0.47–1.47)	0.3 0.14 0.9 0.6 0.5	83.9
CHAARTED [32,33,36]	790	ADT + DTX	ADT	72%	Overall: 57.6 vs 47.2 Subgroup analysis HV: 51.2 vs 34 LV: 63.5 vs NR dn-HV: 48 vs 33 r-HV: 66.9 vs 51.7 dn-LV: 58.3 vs 59.8 r-LV: 69.6 vs NR	0.72 (0.59–0.89) 0.63 (0.50–0.79) 1.04 (0.70–1.55) 0.63 (0.49–0.81) 0.72 (0.36–1.46) 0.86 (0.52–1.42) 1.25 (0.60–2.60)	0.0018 <0.001 0.86 <0.001 0.37 0.55 0.55	53.7
STAMPEDE Arm-C (M1) [107]	1086	ADT + DTX	ADT	95%	Overall: 59.1 vs 43.1 HV: 93.2 vs 76.7 LV: 39.9 vs 35.2	0.81 (0.69–0.95) 0.81 (0.64–1.02) 0.76 (0.54–1.07)	<0.009 0.064 0.107	78.2
LATITUDE [29]	1199	ADT + ABI + PRED	ADT	100%	Overall: 53.3 vs 36.5	0.66 (0.56–0.78)	<0.0001	51.8
STAMPEDE Arm-G (M1) [108]	1002	ADT + ABI + PRED	ADT	95%	M1 subgroup: 79 vs 46	0.60 (0.50–0.71)	<0.0001	6.1 years
ENZAMET [109]	1125	ADT + ENZA	ADT	60%	Overall: NR vs NR Overall OS at 5 years: 67% vs 57%	0.70 (0.58–0.84) Early DTX: 0.82 (0.63–1.06) No early DTX: 0.60 (0.47–0.78) LV: 0.54 (0.39–0.74) HV: 0.79 (0.63–0.98) Synchronous: 0.70 (0.56–0.87) Metachronous 0.71 (0.52–0.98)	<0.0001 (overall)	68
ARCHES [110,111]	1150	ADT + ENZA	ADT	66%	Overall: NR vs NR Subgroup analysis HV: NR vs 45.9 LV: NR vs NR Previous DTX: NR vs NR No previous DTX: NR vs NR	0.66 (0.53–0.81) 0.66 (0.52–0.83) 0.66 (0.43–1.03) 0.74 (0.46–1.20) 0.64 (0.51–0.81)	<0.001 (overall)	44.6
TITAN [112]	1052	ADT + APA	ADT	81%	Overall: NR vs 52.2	0.65 (0.53–0.79) Previous DTX: 1.12 (0.59–2.12) No previous DTX: 0.61 (0.5–0.76)	<0.001 (overall)	44
HORRAD [115]	432	ADT + local RT	ADT	100%	Overall: 45 vs 43	0.90 (0.70–1.14)	0.4	47
STAMPEDE Arm-H [116]	2061	ADT ± DTX + local RT	ADT ± DTX	100%	Overall: 42.5 vs 41.6 LV: 49.5 vs 45.4 HV: 37.6 vs 38.8	0.92 (0.8–1.06) 0.68 (0.52–0.9) 1.07 (0.9–1.28)	0.266 0.007 0.420	37
ARASENS [113]	1306	ADT + DTX + DARO	ADT + DTX	86.1%	Overall: NR vs 48.9 HV: NR vs 42.4 LV: NR vs NR HR: NR vs 43.2 LR: NR vs NR	0.68 (0.57–0.8) 0.69 (0.57–0.82) 0.68 (0.41–1.13) 0.71 (0. 58–0.86) 0.62 (0.4–0.9)	<0.001 (overall)	43.7
PEACE-1 [114]	1173	ADT ± DTX + ABI + PRED (± local RT)	ADT ±DTX (± local RT)	100%	Overall: 5.7 years vs 4.7 years HV: NA LV: NA	0.82 (0.69–0.98) 0.77 (0.62–0.96) 0.93 (0.69–1.28)	0.03 0.019 NA	4.4 years
PEACE-1 [114] (DTX subgroup)	710	ADT + DTX + ABI + PRED (± local RT)	ADT + DTX (± local RT)	100%	Overall: NR vs 4.4 years HV: 5.1 vs 3.5 years LV: NR vs NR	0.75 (0.59–0.95) 0.72 (0.55–0.95) 0.83 (0.50–1.39)	0.017 0.019 0.66	45.6

a Abbreviations: ABI, abiraterone acetate; ADT, androgen deprivation therapy; APA, apalutamide; DARO, darolutamide; dn-HNPC, *de novo* hormone-naive prostate cancer; dn-HV, *de novo* high volume; dn-LV, *de novo* low volume; DTX, docetaxel; ENZA, enzalutamide; HR, hazard ratio; HV, high-volume; LV, low-volume; M, metastatic population; mHNPC, metastatic hormone-naive prostate cancer; NA, not available; NR, not reached; OS, overall survival; PRED, prednisone; r-HNPC, relapsed hormone-naive prostate cancer; r-HV, relapsed high volume; r-LV, relapsed low volume.

Initial trials with docetaxel, such as GETUG-AFU15 [30,31], did not show clear survival advantages, but subsequent studies such as CHAARTED [33,36] and STAMPEDE arm C [107] demonstrated benefits of adding chemotherapy to ADT, particularly in patients with HV disease. Moreover, the implementation of ARTA, including abiraterone acetate and the AR inhibitors apalutamide, darolutamide, and enzalutamide, has shown significant survival advantages in various trials such as LATITUDE [32], STAMPEDE arm G [108], ENZAMET [109], ARCHES [110,111], and TITAN [112]. Recent trials such as ARASENS [113] and PEACE [114] highlight the potential benefits of treatment intensification by combining chemotherapy and AR pathway inhibitors.

The role of radiotherapy to the primary tumor in patients with mHNPC remains a subject of debate, and trials such as HORRAD [115] and STAMPEDE Arm-H [116] have shown varied outcomes depending on disease volume. Although STAMPEDE Arm-H data support the use of radiotherapy in addition to ADT in patients with LV mHNPC, these data were generated before the widespread use of ARTA in mHNPC. Recent data from the PEACE-1 study suggest that the clinical benefit of adding radiotherapy to ADT combined with ARTA is modest at most, and future research should elucidate which patients with mHNPC could benefit from radiotherapy when also receiving ADT and ARTA [114,117].

Refining the criteria for treatment selection remains a challenge because of the lack of validated biomarkers. Although the use of at least one AR-targeting drug seems to be universally indicated, questions arise concerning the indication of docetaxel and radiotherapy for particular patients [33,36,113., 114., 115., 116.]. Moreover, further understanding of the biological effects of intensified therapy on cancer evolution, subclonal selection, and the development of resistance could enable the design of rational, biology-driven clinical trials for testing alternative regimens. For example, an induction period with intense ADT plus AR-targeting drugs followed by ADT alone, or adaptive regimens where treatment is intensified/de-intensified longitudinally based on emerging biomarkers of tumor kinetics and biology, are approaches to be explored by the next generation of clinical trials in mHNPC. Validation of molecularly defined prognostic and predictive biomarkers would also enable trials for testing personalized therapeutic strategies in mHNPC.

**Figure 1:**
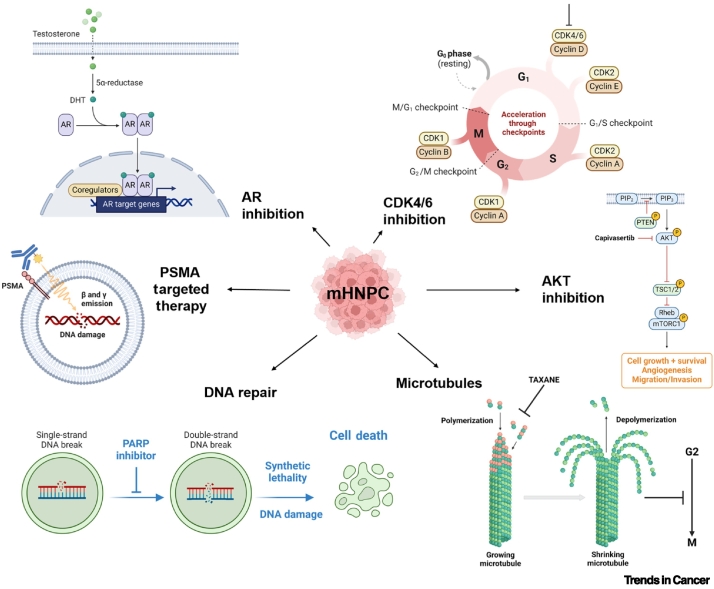
Principal pathways targeted in metastatic hormone-naïve prostate cancer (mHNPC). AR and microtubule targeting are already part of the standard of care, and AKT, DNA repair, PSMA, and CDK4/6 targeting are currently under investigation. Abbreviations: AR, androgen receptor; CDK, cyclin-dependent kinase; PSMA, prostate-specific membrane antigen. Figure created with BioRender.

Furthermore, recent clinical trials focused on novel hormonal agents (NHAs), ADT, and chemotherapy have created the need to study the influence of the treatment schedule (the order and duration of each therapy) in mHNPC antitumor efficacy (NCT05884398, NCT05956639, NCT05676203). In addition, multiple additional ongoing Phase 3 clinical trials are exploring new therapeutic approaches including immunotherapy, radiopharmaceuticals, and molecular targeting agents in this setting (Figure 1 and Table 4).

**Table 4:** Ongoing Phase 3 clinical trials testing new therapeutic approaches for mHNPC^a^, ^b^

Trial	Control arm	Experimental arm	Recruitment status	Population	Primary endpoint(s)
ARANOTE NCT04736199	ADT	ADT + DARO	Active, not recruiting	mHNPC	rPFS
PROSTRATEGY NCT03879122	ADT + DTX (six cycles)	Arm 2: ADT + DTX (six cycles) then nivolumab 3 mg/kg q14 for 1 year Arm 3: ADT + two cycles ipilimumab 3 mg/kg q21, then three cycles DTX, two cycles ipilimumab, three cycles DTX and nivolumab 3 mg/kg q14 for 1 year	Active, not recruiting	mHNPC	OS
PSMAddition NCT04720157	SoC (ARDT + ADT)	7.4 GBq ^177^Lu-PSMA-617 q6w (six cycles) + SoC (ARDT + ADT)	Active, not recruiting	mHNPC	rPFS
CYCLONE-03 NCT05288166	Placebo + ABI + prednisone	Abemaciclib + ABI + prednisone	Active, not recruiting	mHNPC	rPFS
TALAPRO-3 NCT04821622	Placebo + enzalutamide	Talazoparib + enzalutamide	Active, not recruiting	DDR gene mutated mHNPC	rPFS
EvoPAR-PR01 NCT06120491	Placebo + physician's choice NHA (ABI, DARO, or enzalutamide)	Saruparib (AZD5305) + physician's choice NHA (ABI, DARO, or enzalutamide)	Recruiting	mHNPC	rPFS
AMPLITUDE NCT04497844	Placebo + ABI + prednisone	Niraparib + ABI + prednisone	Active, not recruiting	Deleterious germline or somatic HRR gene-mutated mHNPC	rPFS
CAPItello-281 NCT04493853	Placebo + ABI + prednisone	Capivasertib + ABI + prednisone	Active, not recruiting	*PTEN-*deficient mHNPC	rPFS

a Information from ClinicalTrials.gov as per 10/05/2024.

b Abbreviations: ABI, abiraterone; ADT, androgen deprivation therapy; ARDT, androgen receptor-directed therapy; DARO, darolutamide; DDR, DNA damage repair; DTX, docetaxel; GBq, gigabecquerel; HRR homologous recombination repair; NHA, novel hormonal agent; OS, overall survival; PSMA, prostate-specific membrane antigen; q14/q21, every 14/21 days; q6w, every 6 weeks; rPFS, radiographic progression-free survival; SoC, standard of care.

### Immunotherapy

Immunotherapy-based clinical trials in late-stage mCRPC have not so far yielded satisfactory results. Compared to other tumor types, PCa is characterized by an immunologically 'cold' TME that is enriched in immunosuppressive cells [118]. Although the levels of programmed death ligands 1 and 2 (PD-L1 and PD-L2) in PCa cells can vary significantly, preclinical data suggest that treatment with enzalutamide may enhance PD-L1 expression within the TME, potentially fostering immune evasion and resistance [119]. In Phase 2 clinical trials, the combination of antiandrogen therapy and immunotherapy targeting the PD-1/PD-L1 axis has been associated with potentially enhanced and durable response rates in patients with mCRPC that have not responded to enzalutamide, as well as in previously untreated patients [120,121]. Building upon these findings, the KEYNOTE-991 Phase 3 trial (NCT04191096) explored whether this combined treatment approach with enzalutamide plus pembrolizumab was more effective than enzalutamide plus placebo in mHNPC, and stratified patients based on prior docetaxel therapy and the presence of HV disease. Unfortunately, the primary endpoints (overall survival and radiographic progression-free survival) were not met [122,123]. Despite these results, anti-PD1 immunotherapy combined with ADT induced robust immune infiltration in mHNPC [67], and a Phase 2 clinical trial (NCT03951831) investigating standard-of-care chemo-hormonal therapy combined with anti-PD-1 immunotherapy is ongoing.

Inhibiting cytotoxic T lymphocyte-associated protein 4 (CTLA-4) promotes T cell infiltration of the tumor but also triggers upregulation of PD-1 and PD-L1 within the prostate TME [124]. Combined anti-CTLA-4 plus anti-PD1 can partly overcome this adaptive resistance, and this strategy is being evaluated in mHNPC in an ongoing Phase 3 clinical trial (NCT03879122).

Even though immunotherapy has not yet been implemented in mHNPC management, a better understanding of the mHNPC TME features, and of the dynamic changes in the TME upon exposure to hormonal therapy, could lead to new therapeutic opportunities for these patients [67].

## Concluding remarks

**Figure 2:**
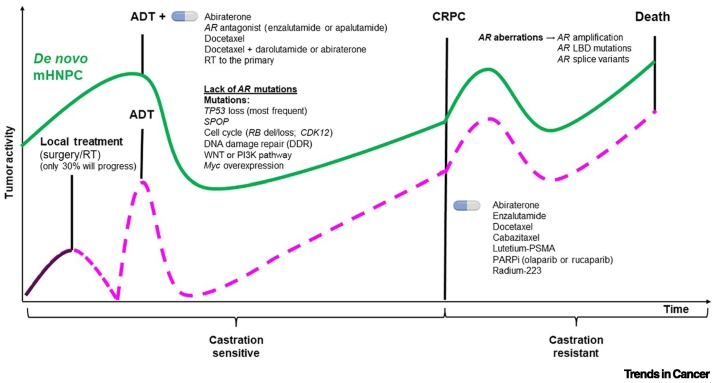
Natural history, molecular alterations, and therapeutic interventions in localized prostate cancer (PCa) and metastatic hormone-naïve prostate cancer (mHNPC). The green line represents the evolution of *de novo* mHNPC, and the purple line represents the progression of localized PCa. Abbreviations: ADT, androgen deprivation therapy; AR, androgen receptor; CRPC, castration-resistant prostate cancer; Del, deletion; LBD, ligand-binding domain; PARPi, PARP inhibitor; PSMA, prostate-specific membrane antigen; RT, radiotherapy.

mHNPC is a lethal form of PCa with distinct biological characteristics and an identifiable patient subgroup based on emerging epidemiological, clinical, and molecular studies. However, the distinct disease course and advances in therapeutic strategies for mHNPC suggest that disease interception before the development of castration resistance is a crucial strategy to improve overall survival (Figure 2). To that end, placing mHNPC in the research limelight is a priority. Recently published and ongoing clinical trials, as well as detailed molecular studies, will provide us with a more comprehensive view of how to diagnose, classify, and treat these patients (see Outstanding questions). Parallel to disease characterization, we need to reinforce the development of experimental models that propel mHNPC research and set the stage for the discovery and validation of stratification and therapeutic innovative strategies. Altogether, advancing this research front will undoubtedly influence PCa survivorship.

Outstanding questions Can we exploit non-invasive biomarkers to aid monitoring and adaptive therapeutic management of mHNPC?

Are there unique molecular features that underlie the development of *de novo* versus relapsed mHNPC, and can they serve as targets for therapeutic innovation?

Are current experimental models of prostate cancer sufficient to understand mHNPC?

Alt-text: Outstanding questions
